# Interactions between Genetic Variants in the *Adiponectin*, *Adiponectin* Receptor 1 and Environmental Factors on the Risk of Colorectal Cancer

**DOI:** 10.1371/journal.pone.0027301

**Published:** 2011-11-07

**Authors:** Li Liu, Rong Zhong, Sheng Wei, Jie-Yun Yin, Hao Xiang, Li Zou, Wei Chen, Ji-Gui Chen, Xia-Wen Zheng, Li-Juan Huang, Bei-Bei Zhu, Quan Chen, Sheng-Yu Duan, Rui Rui, Bei-Fang Yang, Jing-Wen Sun, Duo-Shuang Xie, Yi-Hua Xu, Xiao-Ping Miao, Shao-Fa Nie

**Affiliations:** 1 Department of Epidemiology and Biostatistics, and the Ministry of Education Key Lab of Environment and Health, School of Public Health, Tongji Medical College, Huazhong University of Science and Technology, Wuhan, Hubei, China; 2 Department of Epidemiology and Biostatistics, School of Public Health, Wuhan University, Wuhan, Hubei, China; 3 Department of Surgery, The Eighth Hospital of Wuhan, Wuhan, Hubei, China; 4 Department of Infection Control, Taihe Hospital, Yunyang Medical College, Shiyan, Hubei, China; Rikagaku Kenkyūsho Brain Science Institute, Japan

## Abstract

**Background:**

Metabolic syndrome traits play an important role in the development of colorectal cancer. Adipokines, key metabolic syndrome cellular mediators, when abnormal, may induce carcinogenesis.

**Methodology/Principal Findings:**

To investigate whether polymorphisms of important adipokines, *adiponectin* (*ADIPOQ*) and its receptors, either alone or in combination with environmental factors, are implicated in colorectal cancer, a two-stage case-control study was conducted. In the first stage, we evaluated 24 tag single nucleotide polymorphisms (tag SNPs) across *ADIPOQ* ligand and two *ADIPOQ* receptors (*ADIPOR1* and *ADIPOR2*) among 470 cases and 458 controls. One SNP with promising association was then analyzed in stage 2 among 314 cases and 355 controls. In our study, *ADIPOQ* rs1063538 was consistently associated with increased colorectal cancer risk, with an odds ratio (OR) of 1.94 (95%CI: 1.48–2.54) for CC genotype compared with TT genotype. In two-factor gene-environment interaction analyses, rs1063538 presented significant interactions with smoking status, family history of cancer and alcohol use, with ORs of 4.52 (95%CI: 2.78–7.34), 3.18 (95%CI: 1.73–5.82) and 1.97 (95%CI: 1.27–3.04) for smokers, individuals with family history of cancer or drinkers with CC genotype compared with non-smokers, individuals without family history of cancer or non-drinkers with TT genotype, respectively. Multifactor gene-environment interactions analysis revealed significant interactions between *ADIPOQ* rs1063538, *ADIPOR1* rs1539355, smoking status and BMI. Individuals carrying one, two and at least three risk factors presented 1.18–fold (95%CI:0.89–fold to 1.58–fold), 1.87–fold (95%CI: 1.38–fold to2.54–fold) and 4.39–fold (95%CI: 2.75–fold to 7.01–fold) increased colorectal cancer risk compared with those who without risk factor, respectively (*P*_trend_ <0.0001).

**Conclusions/Significance:**

Our results suggest that variants in *ADIPOQ* may contribute to increased colorectal cancer risk in Chinese and this contribution may be modified by environmental factors, such as smoking status, family history of cancer and BMI.

## Introduction

Colorectal cancer is one of the leading cause of cancer morbidity and mortality, accounting for an estimated 1, 330, 000 new cases and 608, 000 cancer deaths in 2008 worldwide [1]. Colorectal cancer has been long prevalent in western populations and was estimated to cause 142, 570 new cases and 51, 370 cancer deaths in Unite State in 2010 [2]. In the past decades, the incidence rate of colorectal cancer increased remarkably in China. For example, developed area in China such as Shanghai experienced an annual increase of 4.2% in colorectal cancer incidence, which was even significantly higher than the average increase of 2% worldwide during the past twenty years [3].

Although the causation of colorectal cancer has not been completely understood, epidemiological studies found that western dietary and behavior patterns, such as high fat, low fiber intake and deficiency of physical activity, were the main reason for increasing incidence of colorectal cancer in developing countries. The metabolic syndrome, a consequence of western dietary and behavior patterns and characterized by obesity, insulin resistance and hypertension, was subsequently demonstrated to contribute to colorectal cancer risk [4], [5], [6]. Epidemiological studies have reported an increased risk of colorectal cancer in obese individuals compared with normal weight individuals [7], [8]. Similarly, markers of insulin resistance, such as high circulation levels of C-peptide and insulin-like growth factor-binding protein 1(IGFBP-1), were showed to be directly associated with colorectal cancer risk [9], [10]. Meanwhile, the observation of an ecologic correlation between adipokines secretion disorders and metabolic syndrome traits, has spawned a number of epidemiological studies of the association between important adipokines, especially between adiponectin (ADIPOQ) and its receptors (ADIPORs) with obesity and insulin resistance, which showed that circulating ADIPOQ level was significantly negatively, whereas ADIPORs level was positively, associated with metabolic syndrome traits [11], [12].

Given the association between metabolic syndrome traits and colorectal cancer risk and the key role of *ADIPOQ* and its receptor genes in the development of obesity and insulin resistance, emerging interest was focused on the role of *ADIPOQ* and its receptor genes in colorectal carcinogenesis. In vitro, ADIPOQ presented the tendency of growth inhibition and apoptosis induction in colorectal cancer cell lines [13]. In vivo, mice with disruptions in serum ADIPOQ developed more intestinal tumors [14]. Recently, several lines of evidence have demonstrated the inverse association between serum ADIPOQ and colorectal cancer risk [15], [16], [17]. In a prospective, nested case-control study, men in the highest ADIPOQ quintile were found to have a 68% lower risk of colorectal cancer compared with men in the lowest quintile (relative risk [RR], 0.42; 95% confidence interval [CI], 0.23–0.78)[18]. The anticancer role of ADIPOQ was mainly induced by its receptors, which have been demonstrated to repress colon cancer cell lines (including HCT116, HT29 and LoVo) proliferation via ADIPOR1- and -R2-mediated 5′-AMP -activated protein kinase (AMPK). Furthermore, knockdown of *ADIPORs* could relieve the suppressive effect of ADIPOQ on the growth of colon cancer cells [19]. In addition, overexpression of ADIPORs could activate peroxisome proliferator-activated receptor-a (PPAR-a), which was reported to play a role in inhibition of FAS and activation of epidermal growth factor receptor family to promote cancer formation [20], [21]. Moreover, recent epidemiological studies have also detected elevated expression of ADIPORs in colorectal carcinomas than in normal gastrointestinal tissue [22].

Several polymorphisms in *ADIPOQ* and its receptor genes have been demonstrated to influence the expression of these genes and subsequent cancer risk [23], [24], [25]. Kaklamani et al. showed that some polymorphisms of the *ADIPOQ* and its receptor genes were associated with breast cancer [26], prostate cancer [27] and colorectal cancer [28] risk in Caucasian. However, to date, these have been few studies addressing the role of genetic variants in *ADIPOQ* and its receptor genes as cancer susceptibility factors in Chinese population. Therefore, we performed a two-stage case-control study to systemically evaluate single nucleotide polymorphisms (SNPs) of *ADIPOQ* and its receptor genes as a predictor of colorectal cancer risk in Chinese population.

## Results

### Characteristics of the study population

Table 1 lists the characteristics of individuals included in the two-stage case-control study. There were no significant differences in the distribution of age, sex, alcohol use and BMI between cases and controls in either stage. The median age was 58 years old (interquartile range, 48–67 years old) and 56 years old (interquartile range, 46–67 years old) in controls in the first and second stage, respectively, compared with 58 yeas old (interquartile range, 51–67 years old) and 59 yeas old (interquartile range, 49–68 years old) in cases, respectively. More smokers were observed in cases than in controls (odds ratio [OR] = 1.95, 95%CI: 1.56–2.45 in combined analysis). In addition, more cases possessed family history of cancer in both case-control studies (OR = 1.78, 95%CI: 1.34–2.38 in combined analysis).

**Table 1:** Characteristics of including participants in the two-stage case-control study.

	Stage 1				Stage 2				Combined study			
	Cases (n = 470)	Controls (n = 458)	OR(95%CI)	*P*	Cases (n = 314)	Control (n = 355)	OR(95%CI)	*P*	Cases (n = 784)	Controls (n = 813)	OR(95%CI)	*P*
Age (median)	58	58	/	0.72	59	56	/	0.06	58	57	/	0.10
Sex												
Male	261(55.5%)	258(56.3%)	1.00		187(59.6%)	208(58.6%)	1.00		448(57.1%)	466(57.3%)	1.00	
Female	209(44.5%)	200(43.7%)	1.03(0.80–1.34)	0.81	127(40.4%)	147(41.4%)	0.96(0.71–1.31)	0.80	336(42.9%)	347(42.7%)	1.01(0.83–1.23)	0.94
Smoking status												
Never	294(62.6%)	348(77.7%)	1.00		223(71.5%)	288(81.1%)	1.00		517(66.1%)	636(79.2%)	1.00	
Ever	176(37.4%)	100(22.3%)	2.08(1.56–2.79)	5.86×10^-7^	89(28.5%)	67(18.9%)	1.72(1.19–2.46)	0.003	265(33.9%)	167(20.8%)	1.95(1.56–2.45)	4.87×10^−9^
Alcohol use												
Never	310(66.0%)	319(71.4%)	1.00		235(75.6%)	287(81.1%)	1.00		545(69.8%)	606(75.7%)	1.00	
Ever	160(34.0%)	128(28.6%)	1.29(0.97–1.70)	0.08	76(24.4%)	67(18.9%)	1.39(0.96–2.01)	0.08	236(30.2%)	195(24.3%)	1.25(1.08–1.68)	0.01
BMI (Mean ± SD)	23.2± 3.3	22.7±3.2	/	0.06	22.8± 3.3	22.8± 2.9	/	0.90	23.0 ± 3.4	22.8± 3.3	/	0.15
Family history of cancer												
Without	324(77.0%)	371(83.6%)	1.00		266(86.9%)	336(94.6%)	1.00		590(81.2%)	707(88.5%)	1.00	
With	97(23.0%)	73(16.4%)	1.52(1.08–2.13)	0.02	40(13.1%)	19(5.4%)	2.66(1.50–4.70)	0.001	137(18.8%)	92(11.5%)	1.78(1.34–2.38)	6.22×10^−5^

### Risk associated with individual SNP

Since 2 SNPs in *ADIPOR1* and 1 SNP in *ADIPOR2* were failed in the design of PCR primers in the first stage, a total of 24 tag SNPs in *ADIPOQ* and its receptor genes were analyzed and therefore, the cut-off point of *P* value was set as 0.002 for multi-comparison in the first stage. All SNPs fitted the Hardy-Weinberg equilibrium among controls. The frequency of T allele was 0.55 and 0.54 in controls in the first and second stage, compared with 0.46 and 0.46 in cases, respectively. In the first stage, 1 potential association was found (*P*<0.001). The *ADIPOQ* rs1063538 CC genotype was associated with increased colorectal cancer risk, with an OR of 1.94 (95%CI: 1.37–2.74) compared with TT genotype. In the validation study (Stage 2), the rs1063538 was also associated with increased colorectal cancer risk, with an OR of 1.91 (95%CI: 1.23–2.95) for CC vs. TT genotype. Combined analysis of the 2 studies showed that rs1063538 was significantly associated with increased colorectal cancer risk. Individuals carrying rs1063538 CC genotype or C allele presented 1.94–fold (95%CI: 1.48–fold to 2.54–fold) and 1.79–fold (95%CI: 1.43–fold to 2.25–fold) increased colorectal cancer risk compared with those whose carried TT genotype, respectively (Table 2). To test the statistical power of our sample size, we calculated the power to detect an OR of 1.90 at the first error of 0.002 in two-sided test by using a prevalence of 0.55 (prevalence of T allele of rs1063538 in controls). The results were as follow: combined study, power = 0.99; Stage 1, power = 0.94; and Stage 2, power = 0.81. Results for non-significant SNPs are displayed in Table S1.

**Table 2:** Significant SNP associated with colorectal cancer risk.

SNP	Genotype	Stage 1			Stage 2			Combined Study		
		No. (Cases/Controls)	OR(95%CI) a	*P*	No. (Cases/Controls)	OR(95%CI) a	*P*	No. (Cases/Controls)	OR(95%CI) a	*P*
*ADIPOQ*	TT	128/145	1.00		68/107	1.00		196/252	1.00	
rs1063538	CT	172/200	1.02(0.74–1.41)	0.89	152/171	1.34(0.91–1.96)	0.14	324/371	1.14(0.90–1.46)	0.28
	CC	170/104	1.94(1.37–2.74)	1.94×10^−4^	94/76	1.91(1.23–2.95)	0.004	264/180	1.94(1.48–2.54)	1.49×10^−6^
	CT+TT	300/345	1.00		220/278	1.00		520/623	1.00	
	CC	170/104	1.91(1.42–2.57)	1.79×10^−5^	94/76	1.58(1.10–2.25)	0.01	264/180	1.79(1.43–2.25)	4.49×10^−7^
	T allele frequency	0.46/0.55			0.46/0.54			0.46/0.55		

a Adjusted by age, sex, smoking status and alcohol use.

### Two-factor gene-environment interaction and subgroup analyses

To explore the potential interactions between *ADIPOQ* rs1063538 and BMI, smoking status, alcohol use and family history of cancer, we performed two-factor gene-environment interaction analyses by Logistic Regression in combined population. The results are displayed in Table 3. Smokers carrying CC genotype significantly increased colorectal cancer risk when compared with non-smokers carrying TT genotype, with an OR of 4.52 (95%CI: 2.78–7.34). Individuals with family history of cancer harboring CC genotype was also associated with significantly increased colorectal cancer risk, with an OR of 3.18 (95%CI: 1.73–5.82) compared with individuals without family history of cancer carrying TT genotype. Alcohol users with CC genotype presented 1.97–fold (95%CI: 1.27–fold to 3.04–fold) increased colorectal cancer risk compared with never drinkers carrying TT genotype. We also carried out stratified analyses for *ADIPOQ* rs1063538 to explore the role of the polymorphism in different subgroup population. In never smokers, CC genotype significantly increased colorectal cancer risk, with an OR of 1.74 (95%CI: 1.26–2.40) in comparison with TT genotype. Individuals without family history of cancer harboring CC genotype of rs1063538 exhibited a significantly increased risk for colorectal cancer, with an OR of 1.95 (95%CI: 1.44–2.65) compared with TT genotype. In never alcohol users, CC genotype significantly increased colorectal cancer risk, with an OR of 1.87 (95%CI: 1.35–2.58) when compared with TT genotype. Moreover, in subgroup of BMI<25 kg/m^2^, individuals with CC genotype of rs1063538 was significantly associated with increased colorectal cancer risk, with an OR of 2.05 (95%CI: 1.45–2.90) compared with individuals carrying TT genotype (Table 4).

**Table 3:** Two-factor gene-environment interaction analyses by Logistic Regression in combined population.

Variables	OR(95%CI) a	*P* b
rs1063538 × Smoking status		
TT × Never smoking	1.00	
CT × Ever smoking	1.48(1.06–2.05)	0.02
CC × Ever smoking	4.52(2.78–7.34)	1.10×10^−9^
rs1063538 × Alcohol use		
TT × Never drinking	1.00	
CT × Ever drinking	0.72(0.52–1.01)	0.06
CC × Ever drinking	1.97(1.27–3.04)	0.002
rs1063538 × Family history of cancer		
TT × Without family history of cancer	1.00	
CT × With family history of cancer	1.61(1.03–2.53)	0.04
CC × With family history of cancer	3.18(1.73–5.82)	1.85×10^−4^
rs1063538 × BMI		
TT × (BMI<25 kg/m^2^)	1.00	
CT × (BMI≥25 kg/m^2^)	1.05(0.71–1.54)	0.83
CC × (BMI≥25 kg/m^2^)	1.80(1.14–2.85)	0.01

a Adjusted by age, sex, smoking status and alcohol use.

b The cut-off point of *P* value was set as 0.002 for multi-comparison.

**Table 4:** Stratified analysis of the association between *ADIPIQ* rs1063538 and colorectal cancer risk in combined population.

Variables	*ADIPOQ* rs1063538		
	Comparison	OR(95%CI) a	*P* b
Smoking status			
Ever	CT vs TT	0.98(0.57–1.67)	0.93
	CC vs TT	2.81(1.43–5.50)	0.003
Never	CT vs TT	1.21(0.90–1.62)	0.21
	CC vs TT	1.74(1.26–2.40)	0.001
Alcohol use			
Ever	CT vs TT	0.90(0.51–1.60)	0.73
	CC vs TT	2.13(1.08–4.18)	0.03
Never	CT vs TT	1.28(0.96–1.73)	0.10
	CC vs TT	1.87(1.35–2.58)	1.48×10^−4^
Family history of cancer			
Yes	CT vs TT	1.05(0.56–1.99)	0.88
	CC vs TT	2.14(1.01–4.54)	0.05
No	CT vs TT	1.20(0.91–1.58)	0.20
	CC vs TT	1.95(1.44–2.65)	1.69×10^−5^
BMI (kg/m^2^)			
<25	CT vs TT	1.37(1.00–1.87)	0.05
	CC vs TT	2.05(1.45–2.90)	4.63×10^−5^
≥25	CT vs TT	1.06(0.60–1.87)	0.84
	CC vs TT	1.66(0.90–3.07)	0.11

a Adjusted by age, sex, smoking status and alcohol use.

b The cut-off point of *P* value was set as 0.002 for multi-comparison.

### Multifactor gene-environment interactions by Classification and Regression Trees

**Figure 1:**
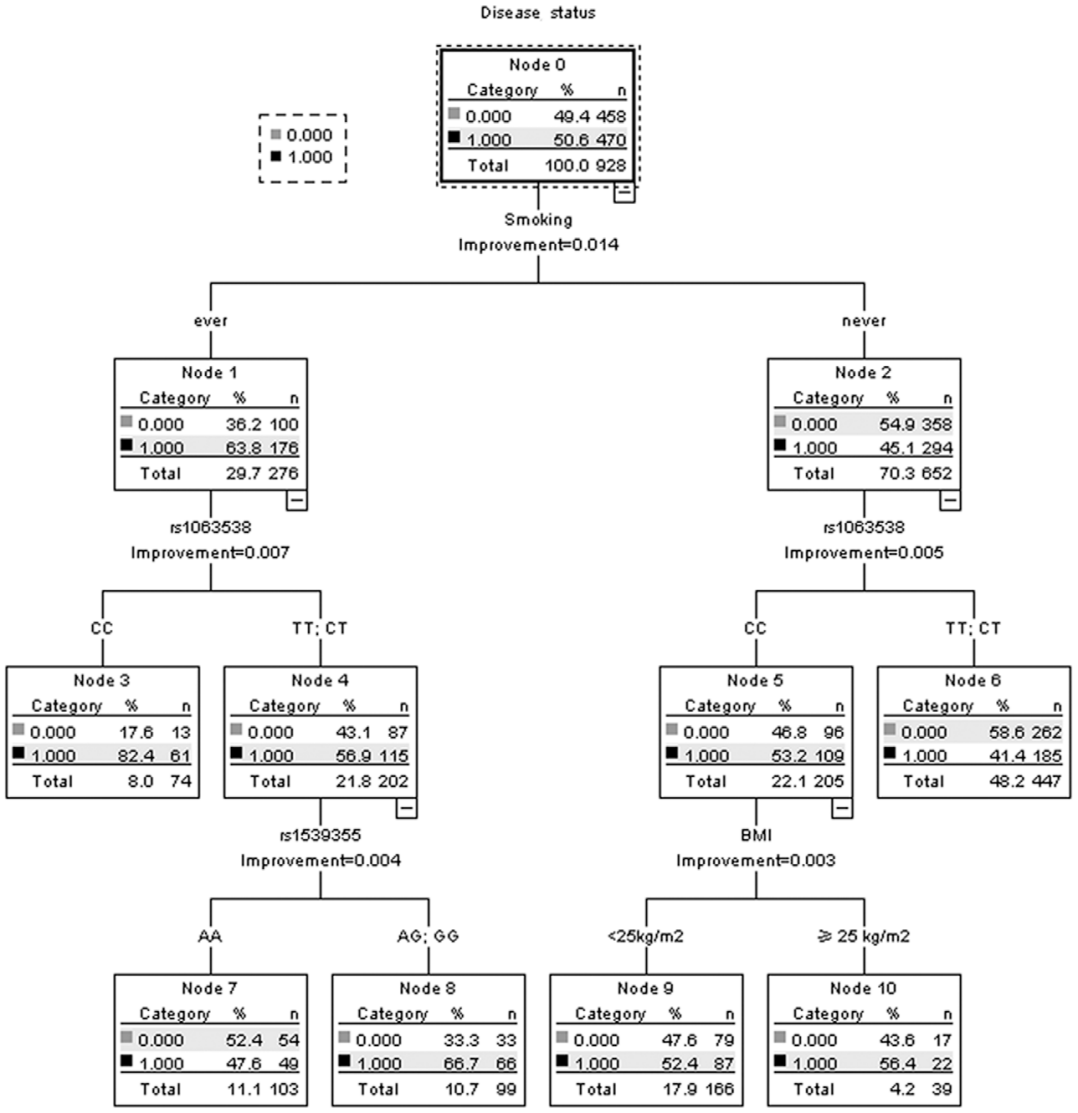
Classification and regression tree analysis of polymorphisms in *ADIPOQ* signaling pathway and environmental factors. Terminal nodes show number of participants in Stage 1. Disease status was classified as cases (1) and controls (0).

We used the data in the first stage to widely explore the potential gene-environment interactions by using Classification and Regression Trees (CART). In the CART analysis, the initial split of the root node was smoking status, and ever smokers had much higher cancer prevalence than never smokers (*P*<0.05), suggesting that smoking status was the strongest factor in colorectal cancer susceptibility. Further inspection of classification tree structure consistently indicated *ADIPOQ* rs1063538 was the strongest split, regardless of smoking status. The combination of ever smoking and rs1063538 CC genotype exhibited the highest risk of colorectal cancer, with a 82.4% patient rate, whereas the combination of never smoking and rs1063538 T allele presented the lowest risk of colorectal cancer, with a 41.4% patient rate. In ever smokers carrying rs1063538 T allele, *ADIPOR1* rs1539355 was the strongest effect associated factor, and the combination of ever smoking, rs1063538 T allele and rs1539355 G allele exhibited a second highest risk of colorectal cancer, with a 66.7% patient rate. In never smokers carrying *ADIPOQ* rs1063538 CC genotype, BMI was the strongest effect associated factor, and the combination of never smoking, rs1063538 CC genotype and non-overweight presented a 52.4% patient rate (Figure 1). (Receiver operating characteristic [ROC] curve in a 10-fold cross-validation for the CART analysis is provided as Figure S1. Area under the curve was 0.62, its 95% CI: 0.59–0.66, *P*<0.001).

### Combined effect of risk factors

To enhance the statistical power, data from combined analysis were used to detect the combined effect of risk factors identified by CART. Since *ADIPOR1* rs1539355 presented interactions with other factors, although the main effect of this polymorphism was not detected in the first stage, we still conducted genotyping for this SNP in Stage 2 (Table S2), which would help to provide data for factor-dosage effect analysis in combined population. To evaluate the combined effect of multifactor associated with colorectal cancer risk, we summed the number of risk factors of smoking status, *ADIPOQ* rs1063538, BMI and *ADIPOR1* rs1539355 in each individual and analyzed the resulting colorectal cancer risk. For environmental factors, smoking and BMI≥25 kg/m^2^ were chosen as risk factors. The genotypes of *ADIPOQ* rs1063538 and *ADIPOR1* rs1539355 were categorized as binary variables according to the split results of CART, namely rs1063538 CC, and rs1539355 AG or GG genotype were viewed as risk factors. We divided the combined population into four subgroups based on the number of risk factors. We found a significant dosage effect association for increased colorectal cancer risk, with an increasing number of risk factors (*P* for trend <0.0001). Compared with individuals without risk factor, individuals carrying 1, 2 and at least 3 risk factors exhibited a gradient of increased colorectal cancer risk with ORs of 1.18 (95%CI: 0.89–1.58), 1.87 (95%CI: 1.38–2.54) and 4.39 (95%CI: 2.75–7.01), respectively (Table 5).

**Table 5:** Cumulative effect of risk factors of smoking, *ADIPOQ* rs1063538, BMI and *ADIPOR1* rs1539355 in colorectal cancer susceptibility in combined study.

No. of risk factors	No. (cases/controls)	OR (95%CI)^a^	*P*	*P* for trend
0	123/197	1.00		<0.0001
1	221/299	1.18(0.89–1.58)	0.25	
2	203/175	1.87(1.38–2.54)	5.38×10^−5^	
≥3	86/32	4.39(2.75–7.01)	5.48×10^−10^	

a Adjusted by age, sex.

## Discussion

This study systematically evaluated the association between a set of polymorphisms in the *ADIPOQ* and its receptor genes and colorectal cancer. The major finding was the significant association of a polymorphism in *ADIPOQ* (rs1063538) with increased colorectal cancer risk. Moreover, the colorectal carcinogenesis role of *ADIPOQ* rs1063538 was modified by environmental factors such as smoking status, family history of cancer, alcohol use and BMI, and the combined effect of multiple potential factors, including smoking, *ADIPOQ* rs1063538, BMI and *ADIPOR1* rs1539355, showed a significant dosage effect in a gene-environment interaction approach.

In our main effect analysis, the *ADIPOQ* rs1063538 was the only SNP exhibited a statistically significant association with increased colorectal cancer risk. This association is biologically plausible. First, the *ADIPOQ* and its receptor genes were newly found to play a role in carcinogenesis especially in obesity-associated malignancies. It has been shown that ADIPOQ could suppress the growth of some malignant cells by regulating AMPK or β-catenin-Wnt pathway, both of which exerted effect in carcinogenesis [29]. ADIPOQ may also contribute to anticancer by promoting apoptosis. ADIPOQ levels have been associated with the activation of apoptotic enzymes in the caspase cascade, which led to cell death, modulation of the expression of several apoptosis-related genes in myelomonocytic cells, and reduction of tumor neovascularization [30]. High level ADIPOQ exposure has been proven to inhibit the proliferation of colorectal cancer cell lines [13], whereas, knockout of *ADIPOQ* could promote tumor growth in mice by reducing macrophage infiltration [31]. Moreover, epidemiological studies have found underexpression of ADIPOQ existing in a wide variety of human cancers, which strongly supported the importance of ADIPOQ in suppression of cancer initiation and development [32]. Second, the SNP rs1063538 is located within the 3'UTR region of *ADIPOQ* and previous studies have demonstrated that polymorphisms in 3'UTR presented significant impact on ADIPOQ level. For example, rs6773957 and rs3774261 were found to be the most strongly associated SNPs in American in a genome-wide linkage and association scans of ADIPOQ level, and both of which are located in the 3'UTR of this gene and captured by rs1063538 [33]. Given the important role of ADIPOQ in regulation of cell proliferation and apoptosis [34], [35], and the role of 3'UTR in regulation of gene expression, it was inferred that polymorphisms in 3'UTR of *ADIPOQ* might play a role in colorectal carcinogenesis, which was documented by Kaklamani et al. [26]. Subsequently, we may infer that rs1063538 could influence colorectal cancer risk by its linkage disequilibrium with other SNPs in the 3'UTR to regulate the expression of *ADIPOQ*, however, it remained to be confirmed. In addition, it is also probable that this SNP is merely a tag SNP which is in linkage disequilibrium with the real causal SNP. The real causal SNP may be located in the coding region and affect the protein function at the posttranslational level [36].

Besides the modest main effect of *ADIPOQ* rs1063538, we also observed significant effect of gene-environment interactions, which were able to amplify the modest effect of the single genetic variant, and enhance the predictive power. In two-factor gene-environment interaction analyses and stratified analyses, we found that individuals carrying *ADIPOQ* rs1063538 CC genotype presented different colorectal cancer risk in different smoking status, family history of cancer categories, alcohol use and BMI. Consistently, a significant interaction was also detected among *ADIPOQ* rs1063538, *ADIPOR1* rs1539355, smoking status and BMI in CART analysis. Although statistical interactions do not necessarily imply biologic interactions, several lines of evidence suggest that our findings may be biologically plausible. As one of the main adipokines, ADIPOQ acts by crosslinking with its receptors, ADIPOR1 and ADIPOR2 [32]. Previously studies have documented that ADIPOQ repressed colon cancer cell lines (including HCT116, HT29 and LoVo) proliferation via ADIPOR1- and -R2-mediated AMPK, whereas, knockdown of ADIPORs such as ADIPOR1 relieved the suppressive effect of ADIPOQ on the growth of cancer cells [19]. Although the *ADIPOR1* rs1539355 was not identified functional SNP, this polymorphism was also found to be associated with reduced insulin resistance, suggesting there might be some linkage between this polymorphism and gene expression or function [37]. Besides, the *ADIPOR1* rs1539355 could highlight the role of *ADIPOQ* SNPs in obesity genotype [38]. Since both insulin resistance and obesity genotypes were demonstrated associated with colorectal cancer risk, it was reasonable to believe the interactions between variants of *ADIPOQ* and its receptor genes played a role in carcinogenesis of colorectal cancer [39]. In addition to the interactions inside *ADIPOQ* signal pathway, the function of *ADIPOQ* and its receptor genes was also modified by some colorectal cancer risk associated environmental factors, such as smoking status and BMI. Previous studies have shown that obesity could potentially influence the activation of *ADIPOQ* and its receptor genes and subsequent cancer risk [40], [41]. For example, Tang et al. indicated that a positive association of genetic variants of *ADIPOQ* with prostate cancer was limited to persons who were overweight [42] and Petridou et al. showed that women with high BMI and low plasma ADIPOQ had 6.5–fold increased risk of endometrial cancer compared with women with normal BMI and higher ADIPOQ concentrations [43]. Smoke exposure was also demonstrated to inhibit the mRNA expression of *ADIPOQ* in adipocytes. When compared with none, environmental tobacco smoke exposure of more than 10 cigarettes per day was associated with low ADIPOQ level [44]. Conversely, smoking cessation was found to be associated with increased plasma ADIPOQ level [45], [46]. Family history of cancer and alcohol use were also established risk factors for colorectal cancer, however, studies for the influence of these two factors in the expression of *ADIPOQ* and its receptors genes were limited. Although there was still some mechanism should be addressed, the interactions might reveal a biological promotion of these factors.

There are some limitations in our study. First, this two-stage case-control study is hospital-based and selection bias may exist, since the controls were from a health examination population which may not be ideal representatives of geographically matched population in similar environmental exposure. However, the controls came from the same region with cases and were randomly sampled, which may reduce the effect of selection bias. Second, the information of patients' BMI is obtained at the first diagnosis of colorectal cancer. Since carcinogenesis is a complex and chronic consumptions, the recent BMI may bring some bias to the association of obesity and colorectal cancer risk. However, a recent large case-control study published in Journal of the National Cancer Institute indicated that BMI based on recent self-reported measures reported similar result with BMI from prospective studies in colorectal cancer risk [47], [48], [49]. Therefore, we think recent BMI in our study will not bring substantial bias to the results of our study. Third, there are some missing data in environmental exposure in both case-control studies, such as family history of cancer, since the participants could not give exact information on related items during interviewing. Therefore, we completed gene-environment interaction analyses in combined population, which may bring more statistical power to the results and reduce bias caused by missing data. Fourth, the sample size is not big enough for some statistical analysis, especially for some subgroup analyses, which may bring some impact to the stability and reliability of the results. So further studies with more sample size, more SNPs and functional explore are warranted to identified the role of *ADIPOQ* gene family and gene-environment interactions in colorectal carcinogenesis.

In summary, this is the first study to systematically assess the impact of germ line genetic variants in *ADIPOQ* and its receptor genes and gene-environment interactions on colorectal cancer risk in Chinese. We found one SNP in *ADIPOQ* (rs1063538) was associated with increased colorectal cancer risk and the potential gene-environment interactions might play more important role in regulating colorectal cancer risk. The identification of novel genetic susceptibility markers of colorectal cancer etiology will not only help us understand the biology of colorectal carcinogenesis but may also be integrated with known clinical, epidemiological and genetic knowledge to help us improve risk prediction of colorectal cancer.

## Materials and Methods

### Ethics statement

The study was informed consent obtained from all final participants and approved by the review board of Tongji Medical College of Huazhong University of Science and Technology.

### Study participants

A two-stage case-control study design was utilized in this investigation. The first case-control study was used to evaluate the *ADIPOQ* and its receptor genes polymorphisms in relation to colorectal cancer risk, and then to validate promising associations in the second independent population. In the first stage, cases were newly diagnosed with colorectal cancer between January 1, 2007 and November 31, 2009 in the Eighth Hospital in Wuhan, without a previous cancer diagnosis, and alive at the time of interview. Recruitment for Stage 2 occurred between January 1, 2009 and August 31, 2010 in Taihe Hospital in Shiyan, and the including criteria were same with cases recruited in Stage 1. Controls were selected from the health examination population in the same hospital during the same period and frequently matched with cases by age (±5 years) and sex in each stage. None of the controls had any personal history of cancer, digestive diseases, hypertension or diabetes at the time of blood donation, which was ascertained with a questionnaire completed by each healthy control. Blood sample and personal information regarding sex, birth year, smoking status, alcohol use, recent weight, height and family history of cancer were collected from each participant, and additional information on age at colorectal cancer diagnosis and clinical characteristics was recorded from cases. Of eligible participants, 470 cases (94.0%) and 458 controls (91.6%), and 314 cases (87.22%) and 355 controls (98.61%) completed in-person interviews and donated blood samples in first and second stage, respectively.

### SNP selection

A total of 100 SNP markers with a minor allele frequency (MAF) ≥0.1 of *ADIPOQ* and its receptor genes were download from HapMap (http://www.hapmap.org/) using phase 1 and phase 2 Data Release 24 (Build 36.3) for the Chinese population (Chinese Han from Beijing–CHB). Tag SNPs were chosen for each gene by using Tagger in Haploview (http://www.broadinstitute.org/haploview/haploview). We used the pair-wise mode and selected a minimal set of markers, such that all alleles to be captured would be correlated at an r^2^≥0.8 with a marker in that set. The *ADIPOQ*, *ADIPOR1* and *ADIPOR2* yielded 10, 9 and 8 tag SNPs, respectively. (Detailed information is displayed in Table S1).

### Polymorphism analysis

Genomic DNA from peripheral blood samples was isolated using Blood Genomic DNA Purification kit (Tiangen Biotech, Beijing, China) following the manufacturer's protocol. Genotyping was carried out in two phases.

In the first stage, the Sequenom MassARRAY platform (Sequenom San diego, CA, USA) was used for high throughput genotyping and assay design [50]. Genotyping was carried out by using iPLEX chemistry on a matrix assisted laser desorption/ionization time-of-flight (MALDI-TOF) mass spectrometer. Multiplex SNP assays were designed by using SPECTRODESIGNER software. PCR reactions, cycling conditions and post-PCR extension reactions were performed as manufacturer's protocol. The iPLEX reaction products were treated with the SpectroCLEAN (Sequenom) resin to remove salts and spotted on a 384 SpectroChip, and then processed and analyzed by the MALDI-TOF mass spectrometer. Genotypes were called using MassARRAY Typer 4.0 software [51]. For each 384-well plate, 20 samples were duplicated and 4 wells were filed with H_2_O (blank) to cross-check contamination and reliability of the system. A whole plate was considered to have failed if: (i) no SNPs had passed call rate of 80%; and/or (ii) if the success rate of duplicate checks was <99.5% and that of the blank <90%; and/or (iii) the success rate of blank check alone had been <75%. SNPs were removed from the analysis when: (i) they were not call in at least 80% of patients and controls; and/or (ii) they were monomorphic, as these are uninformative; and/or (iii) their genotype frequencies deviated from Hardy-Weinberg equilibrium (HWE) expectation, likely due to genotyping errors [52].

In the second stage, polymorphisms were assessed using the 5′-nuclease (Taqman) assay (Applied Biosystems, Foster City, CA, USA). Primers and probes were designed by Primer Express 3.0 (Applied Biosystems). Reactions were completed and read in a 7900 HT TaqMan sequence detector system (Applied Biosystems). Amplification mixture (12.5 µl) of polymerase chain reaction contained 50 ng of DNA, 900 nM of each forward and reverse primer, 300 nM of each specific probe, and 6.25 µl of Taqman Universal PCR Master Mix (Applied Biosystems, Foster City, CA). Amplification was done under the following conditions: 95°C for 10 min followed by 45 cycle of 94°C for 30 s and 62°C for 1 min. Data were analyzed using Allelic Discrimination Program (Applied Biosystems). The call rate was 99.2%. A total of 10% samples were genotyped in duplicate and showed 100% concordance.

### Statistical analysis

Pearson's χ^2^ test was used to compare the differences in distribution of categorical variables (sex, smoking status, alcohol use and family history of cancer) and either Wilcoxon rank-sum test or Student's *t*-test was used for continue variables (age, body weight index [BMI]), where appropriate. In this study, BMI was categorized as overweight (BMI≥25 kg/m^2^) and non-overweight (BMI<25 kg/m^2^)[53]. Individuals who had smoked at less 100 cigarettes in their lifetimes were defined as smokers, and the rest were called non-smokers [54]. Hardy–Weinberg equilibrium was tested by a goodness-of-fit χ^2^ test to compare the observed genotype frequencies to the expected genotype frequencies in controls.

For the main effect of SNPs, unconditional Logistical Regression was conducted to calculate odds ratios (ORs) and their corresponding 95% confidence intervals (CIs), adjusted for potential confounders (age, sex, smoking status and alcohol use). Two-factor gene-environment interaction analyses were also carried out by unconditional Logistical Regression to assess the interactions between polymorphisms and environmental factors, such as BMI, smoking status, alcohol use and family history of cancer. Further stratified analyses were used to explore the role of the associated polymorphisms in different subgroup population. All statistical analyses were two sided. All statistical analyses were performed using SPSS software 12.0 (SPSS, Inc., Chicago, III).

Classification and Regression Trees (CART) analysis was performed by SPSS software to build a decision tree via recursive partitioning in the splitting criteria of Gini index, which depicts how well each genotype or environmental factor variable predicts class. A CART tree is constructed by splitting a node into two child nodes repeatedly, beginning with the root node that contains the whole learning sample. This process continues until the classification reaches the lowest cross-validation error in the terminal node. Subgroups of individuals with differential result of case-control status are identified in the different terminal nodes of the tree, indicating potential presence of interactions [55]. To quantify the cumulative effect of gene-environment interactions, we tailed the total number of risk factors for each individual and set subject without risk factor as the reference group. Colorectal cancer risk for individuals with different number of risk factors was estimated by calculating ORs and 95% CIs using unconditional Logistic Regression after adjustment for age, sex.

## Supporting Information

Figure S1 **Receiver operating characteristic (ROC) curve in a 10-fold cross-validation for the CART analysis.**
(TIF)

Table S1 **Non-significant SNPs associated with colorectal cancer risk in Stage 1**
(DOC)

Table S2 **The association between** ***ADIPOR1*** **rs1539355 and colorectal cancer risk in Stage 2**
(DOC)
