# Supplementary figures and images for: Interactions between Genetic Variants in the Adiponectin, Adiponectin Receptor 1 and Environmental Factors on the Risk of Colorectal Cancer
Source: PLoS One. 2011 Nov 7;6(11):e27301. doi: 10.1371/journal.pone.0027301 (PMC3210156; doi:10.1371/journal.pone.0027301)

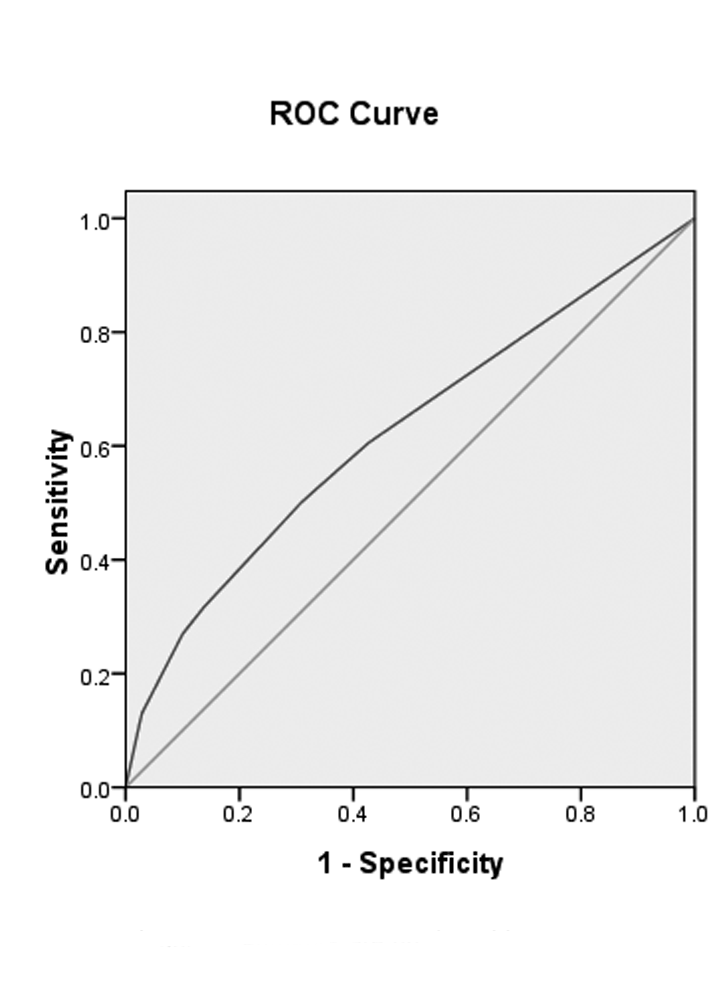

Supplement: Figure S1 — Receiver operating characteristic (ROC) curve in a 10-fold cross-validation for the CART analysis. (TIF) [file pone.0027301.s001.tif]
