# Supplementary material for: Interactions between Genetic Variants in the Adiponectin, Adiponectin Receptor 1 and Environmental Factors on the Risk of Colorectal Cancer
Source: PLoS One. 2011 Nov 7;6(11):e27301. doi: 10.1371/journal.pone.0027301 (PMC3210156; doi:10.1371/journal.pone.0027301)
Supplement: Table S1 — Non-significant SNPs associated with colorectal cancer risk in Stage 1 (DOC) [file pone.0027301.s002.doc]

Table S 1 Non-significant SNPs associated with colorectal cancer risk in Stage 1

| Gene | SNP | Genotype | No. (Case/Control) | OR(95%CI) ^a^ | *P*^b^ |
| --- | --- | --- | --- | --- | --- |
| *ADIPOQ* | rs3774262 | GG | 235/225 | 1.00 |  |
|  |  | GA | 194/196 | 0.98(0.74–1.29) | 0.89 |
|  |  | AA | 38/36 | 0.97(0.59–1.59) | 0.90 |
| *ADIPOQ* | rs822391 | TT | 368/365 | 1.00 |  |
|  |  | TC | 90/88 | 1.09(0.77–1.52) | 0.63 |
|  |  | CC | 7/5 | 1.38(0.43–4.48) | 0.59 |
| *ADIPOQ* | rs7649121 | AA | 269/270 | 1.00 |  |
|  |  | AT | 149/153 | 0.95(0.71–1.27) | 0.73 |
|  |  | TT | 46/34 | 1.32(0.81–2.14) | 0.27 |
| *ADIPOQ* | rs12495941 | GG | 163/161 | 1.00 |  |
|  |  | GT | 225/207 | 1.11(0.83–1.49) | 0.50 |
|  |  | TT | 78/87 | 0.89(0.61–1.31) | 0.56 |
| *ADIPOQ* | rs1063539 | GG | 273/272 | 1.00 |  |
|  |  | GC | 166/161 | 1.09(0.82–1.45) | 0.55 |
|  |  | CC | 22/20 | 1.06(0.56–2.01) | 0.86 |
| *ADIPOQ* | rs16861194 | AA | 339/323 | 1.00 |  |
|  |  | AG | 111/117 | 0.90(0.66–1.22) | 0.50 |
|  |  | GG | 14/18 | 0.72(0.35–1.49) | 0.38 |
| *ADIPOQ* | rs1501299 | CC | 262/226 | 1.00 |  |
|  |  | CA | 167/201 | 0.71(0.54–0.94) | 0.02 |
|  |  | AA | 36/29 | 0.98(0.57–1.66) | 0.93 |
| *ADIPOQ* | rs266729 | CC | 237/240 | 1.00 |  |
|  |  | CG | 184/179 | 1.02(0.77–1.34) | 0.91 |
|  |  | GG | 44/37 | 1.20(0.74–1.95) | 0.45 |
| *ADIPOQ* | rs182052 | GG | 138/149 | 1.00 |  |
|  |  | GA | 240/210 | 1.23(0.91–1.67) | 0.18 |
|  |  | AA | 89/98 | 0.95(0.65 –1.38) | 0.78 |
| *ADIPOR1* | rs10920532 | GG | 278/304 | 1.00 |  |
|  |  | GA | 163/139 | 1.24(0.93–1.65) | 0.14 |
|  |  | AA | 26/15 | 1.90(0.97–3.75) | 0.06 |
| *ADIPOR1* | rs1342387 | GG | 189/165 | 1.00 |  |
|  |  | GA | 222/227 | 0.87(0.65–1.15) | 0.33 |
|  |  | AA | 56/64 | 0.77(0.50–1.17) | 0.22 |
| *ADIPOR1* | rs7539542 | GG | 172/180 | 1.00 |  |
|  |  | GC | 219/218 | 1.03(0.77–1.37) | 0.87 |
|  |  | CC | 78/60 | 1.33(0.88–2.01) | 0.17 |
| *ADIPOR1* | rs1539355 | AA | 244/267 | 1.00 |  |
|  |  | AG | 181/169 | 1.16(0.88–1.53) | 0.31 |
|  |  | GG | 43/22 | 2.08(1.19–3.63) | 0.01 |
| *ADIPOR1* | rs12045862 | TT | 147/149 | 1.00 |  |
|  |  | TC | 211/213 | 1.00(0.73–1.35) | 0.97 |
|  |  | CC | 111/93 | 1.15(0.80–1.66) | 0.46 |
| *ADIPOR1* | rs10581 | CC | 383/377 | 1.00 |  |
|  |  | CT | 84/76 | 1.05(0.74–1.48) | 0.80 |
|  |  | TT | 2/3 | 0.50(0.08–3.16) | 0.46 |
| *ADIPOR1* | rs2275737 | CC | 314/323 | 1.00 |  |
|  |  | CA | 144/122 | 1.21(0.92–1.61) | 0.17 |
|  |  | AA | 11/13 | 0.84(0.38–1.88) | 0.67 |
| *ADIPOR2* | rs10773989 | TT | 155/139 | 1.00 |  |
|  |  | TC | 231/209 | 0.89(0.65–1.20) | 0.43 |
|  |  | CC | 77/106 | 1.05(0.72–1.54) | 0.78 |
| *ADIPOR2* | rs1029629 | AA | 209/191 | 1.00 |  |
|  |  | AC | 188/209 | 0.82(0.62–1.09) | 0.18 |
|  |  | CC | 69/57 | 1.07(0.71–1.62) | 0.73 |
| *ADIPOR2* | rs11061973 | GG | 241/241 | 1.00 |  |
|  |  | GA | 174/179 | 1.03(0.77–1.36) | 0.86 |
|  |  | AA | 52/36 | 1.43(0.89–2.28) | 0.14 |
| *ADIPOR2* | rs10773983 | GG | 153/142 | 1.00 |  |
|  |  | GA | 206/227 | 0.89(0.65–1.20) | 0.43 |
|  |  | AA | 98/85 | 1.05(0.72–1.54) | 0.79 |
| *ADIPOR2* | rs11612414 | GG | 372/377 | 1.00 |  |
|  |  | GA | 95/79 | 1.23(0.88–1.73) | 0.22 |
|  |  | AA | 2/2 | 1.05(0.14–7.76) | 0.96 |
| *ADIPOR2* | rs11061980 | AA | 377/373 | 1.00 |  |
|  |  | AC | 86/80 | 1.06(0.75–1.49) | 0.74 |
|  |  | CC | 5/4 | 1.26(0.33–4.80) | 0.74 |
| *ADIPOR2* | rs11061932 | AA | 288/306 | 1.00 |  |
|  |  | AG | 167/131 | 1.35(1.02–1.80) | 0.04 |
|  |  | GG | 15/21 | 0.79(0.40–1.60) | 0.52 |

^a^ Adjusted by age, sex, smoking status and alcohol use

^b^ The cut-off point of *P* value was set as 0.002 for multi-comparison

Note: The design of PCR primers of rs16850797 and rs16850799 in *ADIPOR1* and rs2108642 in *ADIPOR2* was failed in case-control 1.
