# Supplementary material for: Interactions between Genetic Variants in the Adiponectin, Adiponectin Receptor 1 and Environmental Factors on the Risk of Colorectal Cancer
Source: PLoS One. 2011 Nov 7;6(11):e27301. doi: 10.1371/journal.pone.0027301 (PMC3210156; doi:10.1371/journal.pone.0027301)
Supplement: Table S2 — The association between ADIPOR1 rs1539355 and colorectal cancer risk in Stage 2 (DOC) [file pone.0027301.s003.doc]

Table S 2 The association between ADIPOR1 rs1539355 and colorectal cancer risk in Stage 2

| Gene | SNP | Genotype | No. (Case/Control) | OR(95%CI) ^a^ | *P* |
| --- | --- | --- | --- | --- | --- |
| *ADIPOR1* | rs1539355 | AA | 156/187 | 1.00 |  |
|  |  | AG | 130/133 | 1.18(0.85–1.64) | 0.33 |
|  |  | GG | 23/30 | 0.84(0.46–1.52) | 0.56 |

^a^ Adjusted by age, sex, smoking status and alcohol use
